# Substrate Specificity Analysis of Dihydrofolate/Dihydromethanopterin Reductase Homologs in Methylotrophic α-Proteobacteria

**DOI:** 10.3389/fmicb.2018.02439

**Published:** 2018-10-11

**Authors:** Mark Burton, Chidinma Abanobi, Kate Tzu-Chi Wang, Yihua Ma, Madeline E. Rasche

**Affiliations:** Department of Chemistry and Biochemistry, Center for Applied Biotechnology Studies, California State University, Fullerton, Fullerton, CA, United States

**Keywords:** methylotrophic bacteria, dihydrofolate reductase, dihydromethanopterin reductase, methanopterin, one-carbon transfer

## Abstract

Methane-producing archaea and methylotrophic bacteria use tetrahydromethanopterin (H_4_MPT) and/or tetrahydrofolate (H_4_F) as coenzymes in one-carbon (C1) transfer pathways. The α-proteobacterium *Methylobacterium extorquens* AM1 contains a dihydromethanopterin reductase (DmrA) and two annotated dihydrofolate reductases (DfrA and DfrB). DmrA has been shown to catalyze the final step of H_4_MPT biosynthesis; however, the functions of DfrA and DfrB have not been examined biochemically. Moreover, sequence alignment (BLAST) searches have recognized scores of proteins that share up to 99% identity with DmrA but are annotated as diacylglycerol kinases (DAGK). In this work, we used bioinformatics and enzyme assays to provide insight into the phylogeny and substrate specificity of selected Dfr and DmrA homologs. In a phylogenetic tree, DmrA and homologs annotated as DAGKs grouped together in one clade. Purified histidine-tagged versions of the annotated DAGKs from *Hyphomicrobium nitrativorans* and *M. nodulans* (respectively, sharing 69 and 84% identity with DmrA) showed only low activity in phosphorylating 1,2-dihexanoyl-*sn*-glycerol when compared with a commercial DAGK from *Escherichia coli.* However, the annotated DAGKs successfully reduced a dihydromethanopterin analog (dihydrosarcinapterin, H_2_SPT) with kinetic values similar to those determined for *M. extorquens* AM1 DmrA. DfrA and DfrB showed little or no ability to reduce H_2_SPT under the conditions studied; however, both catalyzed the NADPH-dependent reduction of dihydrofolate. These results provide the first evidence that DfrA and DfrB function as authentic dihydrofolate reductases, while DAGKs with greater than 69% identity to DmrA may be misannotated and are likely to function in H_4_MPT biosynthesis.

## Introduction

In the facultative methylotroph *Methylobacterium extorquens* AM1, growth on single-carbon (C_1_) substrates involves the use of both tetrahydromethanopterin (H_4_MPT) and tetrahydrofolate (H_4_F) (Chistoserdova et al., 1998). H_4_MPT was initially thought to be exclusive to methanogenic archaea and sulfur-dependent hyperthermophilic archaea (Achenbach-Richter et al., 1987; DiMarco et al., 1990; Gorris et al., 1991). However, the discovery of H_4_MPT-linked C_1_ transfer enzymes in the Bacteria domain has provided evidence for the use of H_4_MPT beyond a methane-generating pathway (Chistoserdova et al., 1998; Vorholt et al., 1999; Chistoserdova et al., 2004; Chistoserdova, 2016). In the aerobic α-proteobacterium *M. extorquens* AM1, methylotrophy involves the use of dephospho-H_4_MPT in a series of oxidative steps to catabolize reduced C_1_ compounds to CO_2_ (Chistoserdova et al., 1998); this is in contrast to the reduction of CO_2_ to methane in the anaerobic metabolism of methanogenic archaea (DiMarco et al., 1990). The use of methylotrophs in biotechnology has gained interest because of its application to the microbial production of useful industrial chemicals starting with C_1_ compounds as an alternative to glucose and other conventional sugar or acid substrates (Schrader et al., 2009; Ochsner et al., 2015).

In the pathways of H_4_MPT and H_4_F biosynthesis, the last step requires the activity of dihydromethanopterin reductase (Dmr) or dihydrofolate reductase (Dfr). *M. extorquens* AM1 contains one dihydromethanopterin reductase (DmrA) and two putative dihydrofolate reductases, DfrA and DfrB, that, respectively, share 26% identity (41% similarity) and 34% identity (53% similarity) with DmrA. The *dmrA* gene was first discovered using transposon mutagenesis (Marx et al., 2003) and later deletion mutagenesis which produced a phenotype similar to that of mutants with deletions in H_4_MPT biosynthesis genes (Marx et al., 2003; Rasche et al., 2004; Chistoserdova et al., 2005). Homology of DmrA to dihydrofolate reductases led to the proposal that DmrA evolved from an ancestral dihydrofolate reductase following horizontal transfer of H_4_MPT biosynthesis genes from anaerobic archaea to aerobic bacteria (Marx et al., 2003). A driving force for the evolution of DmrA from dihydrofolate reductase may have been the lack of archaea-specific electron donors such as Factor-420 in the recipient bacteria. Absence of a corresponding archaeal electron donor could render the dihydromethanopterin reductase useless in bacteria, providing selective pressure to modify the substrate specificity of an NADPH-dependent dihydrofolate reductase to reduce dihydromethanopterin (Marx et al., 2003; Caccamo et al., 2004).

**FIGURE 1:**
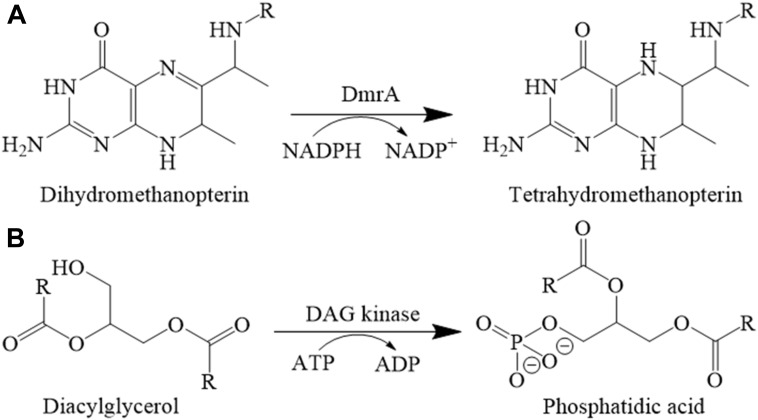
Reactions catalyzed by **(A)** DmrA and **(B)** DAGK.

DmrA has been shown to catalyze the final step of H_4_MPT biosynthesis in *M. extorquens* AM1 (Caccamo et al., 2004) (**Figure 1A**); however, DmrA shares no sequence homology with the FMN-containing dihydromethanopterin reductase discovered in archaea (DmrX) or related archaeal-like flavoproteins (AfpA and DmrB) from β-proteobacteria (Kalyuzhnaya et al., 2005; McNamara et al., 2014; Wang et al., 2014). The FMN prosthetic groups of DmrX and AfpA/DmrB appear to be critical for electron transfer (McNamara et al., 2014; Wang et al., 2014) and may contribute to the absence of homology with the NADPH-dependent DmrA, which lacks flavin cofactors.

In *M. extorquens*, the dihydrofolate reductase homologs DfrA and DfrB have not been examined biochemically. When originally discovered, a role for DfrA in the synthesis of H_4_F was proposed based on its 50% sequence identity to dihydrofolate reductase from *Lactobacillus casei* (Marx et al., 2003) and the genomic location of *dfrA* near the H_4_F synthesis genes *folC* and *folE* in *M. extorquens* (Chistoserdova et al., 2003). Furthermore, the *dfrA* gene is located directly downstream of a gene encoding a putative H_4_F-dependent thymidylate synthase (Marx et al., 2003).

**FIGURE 2:**
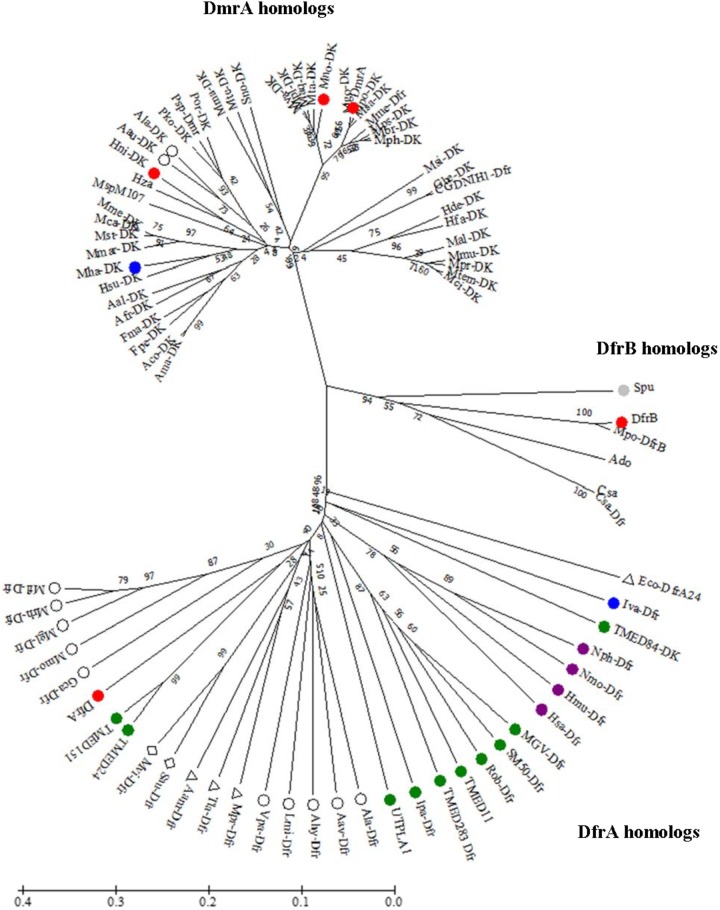
Unrooted phylogenetic tree showing position of DfrB (AY093433) relative to orthologs including DfrA (AY093432) and DmrA (AY093431). Sequences were aligned using EMBL-EBI OMEGA bioinformatics tools with default parameters. Phylogenetic analysis was performed by using Maximum Likelihood method with 10,000 bootstrap replicates (Felsenstein, 1985) within MEGA7 software (Kumar et al., 2016). Abbreviation DAGK (DK). Color designations are Firmicute (blue), Actinobacteria (gray), Planctomycetes (green), Euryarchaeota (purple), β-proteobacteria (open circle), γ-proteobacteria (triangle), and δ-proteobacteria (diamond). DfrA, DfrB, DmrA, Mno-DK, and Hni-DK (red). The bar denotes 1 estimated substitution per 100 amino acid positions.

Little is known about the function of DfrB. When we conducted a BLAST search using *M. extorquens* DfrB as the sequence alignment query, only a few homologs with high sequence identity could be identified. In a phylogenetic tree, these clustered together as a single group (**Figure 2**). Among the more distantly related homologs, one clade included DfrA and numerous annotated dihydrofolate reductases (30–48% identical to DfrB). The last clade consisted of a few known DmrA sequences (34–42% identical to DfrB) and a large number of proteins annotated as diacylglycerol kinases (DAGKs) but sharing 60–99% identity with DmrA from *M. extorquens*. This is curious because DAGKs function in phosphorylation reactions rather than in the reduction of pterins, as shown in **Figure 1B**. To provide insight into possible roles of the DmrA, DfrA, and DfrB homologs, we have used bioinformatics to assess phylogenetic relationships among the homologs and enzyme assays to probe biochemical function.

## Materials and Methods

### Bioinformatics

The DfrB nucleotide sequence (GenBank no. AY093433) (Marx et al., 2003) was used as the query in a non-redundant database BLASTx (translated nucleotide to protein) in the National Center for Biotechnology Information Database (NCBI) using default algorithm patterns with the exception of limiting to 5,000 maximum target sequences (Altschul et al., 1997). Similar results were obtained using the DfrB protein sequence in BLASTp. Sequences were aligned using the Clustal Omega program (Sievers et al., 2011; McWilliam et al., 2013; Li et al., 2015). Aligned sequences were analyzed for phylogenetic relationships and unrooted tree construction (Kumar et al., 2016). The String v10.5 database was used to assess the gene/protein-protein relationships of the neighboring genes to *dfrA* and *dmrA* (Snel et al., 2000; von Mering et al., 2003, 2005, 2007; Jensen et al., 2009; Szklarczyk et al., 2011, 2015, 2017; Franceschini et al., 2013, 2016) and BPROM operon predictive method was applied to these genes (Li, 2011). Neighboring genes without any clear annotations in subsequent sequence alignment searches were analyzed using the Protein Homology/analogy Recognition Engine v2.0 database (Kelley et al., 2015).

### Chemicals

Luria-Bertani/Miller broth (LB) (Becton, Dickinson and Company, Franklin Lakes, NJ) was purchased from Thermo Fisher Scientific (Waltham, MA, United States). Tris(hydroxymethyl)aminomethane (Tris), 1,4-piperazinediethanesulfonic acid (PIPES), dibasic sodium phosphate (Na_2_HPO_4_), monobasic potassium phosphate (KH_2_PO_4_), D-(+)-glucose, magnesium sulfate (MgSO_4_), β-mercaptoethanol (2-ME), kanamycin sulfate, imidazole, sodium ascorbate, magnesium acetate, and ammonium chloride (NH_4_Cl) were also from Thermo Fisher. Isopropyl-β-D-thiogalactopyranoside (IPTG) was from Ubiquitin-Proteasome Biotechnologies (UBP-Bio, Aurora, CO). N-[Tris(hydroxyl)methyl]-2-aminoethanesulfonic acid (TES), 3-(morpholino)propanesulfonic acid (MOPS), sodium acetate, dihydrofolate (H_2_F), NADH, NADPH, ethylenediaminetetraacetic acid (EDTA), ethylene glycol-bis(β-aminoethyl ether)-*N,N,N*′*,N*′-tetraacetic acid (EGTA), phosphoenolpyruvate (PEP), adenosine 5′-triphosphate (ATP), lithium chloride (LiCl), and deoxyribonuclease I (DNase I) bovine were from Sigma-Aldrich (St. Louis, MO, United States). 1,2-dihexanoyl-*sn*-glycerol was from Cayman Chemical (Ann Arbor, MI, United States). Gasses were from Airgas (Placentia, CA, United States). Unless otherwise noted, all other chemicals were purchased from Thermo Fisher Scientific.

### Gene Synthesis and Transformation

The *dfrA* and *dfrB* genes were subcloned with an N-terminal six-histidine (H_6_) tag into the NdeI and BamHI sites of the pET-41a(+) expression vector (Novagen, Madison, WI, United States) by GenScript (Piscataway, NJ, United States). For production of H_6_-DfrA or H_6_-DfrB, the corresponding plasmid was transformed into chemically competent BL21(DE3) cells (Stratagene, La Jolla, CA, United States). Similarly, cell lines were created to produce DmrA (BL21 + pET41a: H_6_-DmrA or pET41a:DmrA-H_4_) and the annotated DAGKs from *Hyphomicrobium nitrativorans* and *M. nodulans* (BL21 + pET41a: Hni-DAGK-H_6_) or BL21 + pET41a:Mno-DAGK-H_6_). The estimated molecular masses of the corresponding histidine-tagged proteins are 19.3 kDa for H_6_-DfrA, 19.4 kDa for H_6_-DfrB, 15.8 kDa for DmrA-H_4_, and 16.2 kDa for both Hni-DAGK-H_6_ and Mno-DAGK-H_6_.

### Cell Growth and Gene Induction

For the production of H_6_-DmrA and DmrA-H_4_, an overnight culture of BL21 cells with pET41a: H_6_-DmrA or pET41a: DmrA-H_4_ was used to inoculate 1 liter of a modified M9 minimal medium (Sambrook and Russell, 2001) containing 48 mM Na_2_HPO_4_, 22 mM KH_2_PO_4_, 19 mM NH_4_Cl, and 17 mM NaCl [pH 7.4], supplemented with 0.4% (w/v) D-(+)-glucose, 2 mM MgSO_4_, and kanamycin (50 μg/ml). Cells were grown at 37°C with shaking (180 rpm). When the optical density at 600 nm reached approximately 0.4, the cells were transferred to another platform shaker previously equilibrated to 15°C (180 rpm) for approximately 45 min. When the optical density at 600 nm reached 0.55–0.60, gene expression was induced with IPTG to 1 mM. The cells were grown at 15°C for 16 h, and then the cell suspension was centrifuged (5,000 × *g*, 15 min, 4°C). The cell pellet was washed in 30 ml of 50 mM TES [pH 8.0], collected by centrifugation (7,000 ×*g*, 15 min, 4°C), and stored at -20°C.

To produce tagged DfrA, DfrB, *M. nodulans* DAGK, and *H. nitrativorans* DAGK proteins (H_6_-DfrA, H_6_-DfrB, Mno-DAGK-H_6_, and Hni-DAGK-H_6_), overnight cultures of BL21 cells with the appropriate plasmid were used to inoculate 1 L of LB medium (Bertani, 1951) containing kanamycin (50 μg/ml). Cells were grown at 37°C with shaking (180 rpm). For H_6_-DfrA, H_6_-DfrB, and Hni-DAGK-H_6_, when the optical density at 600 nm reached approximately 0.6, gene expression was induced with IPTG to 1 mM. The culture was transferred to a platform shaker at 20°C, and cells were grown for 16 h with shaking (180 rpm). For Mno-DAGK-H_6,_ after induction, the culture was grown at 30°C for 6 h with shaking (180 rpm). All cells were collected by centrifugation (5,000 ×*g*, 15 min, 4°C), washed with 30 ml of 50 mM TES, pH 8, centrifuged (7,000 ×*g*, 15 min, 4°C), and stored at -20°C.

### Cell Lysis and Protein Purification

All cells were lysed at 20,000 lb/in^2^ by one pass through a cold French Press cell (Thermo Fisher Corporation, Waltham, MA, United States) at 4°C in 50 mM Tris, 200 mM NaCl, 20 mM imidazole, 15 mM 2-ME [pH 8.0], and 2 μL of DNase I. Lysed cells were centrifuged for 1 h at 4°C (32,000 ×*g*). The supernatant (cell-free extract, CFE) was removed and centrifuged for an additional 15 min. The CFE was incubated with 1-part Nickel Nitrilotriacetic acid resin (NiNTA, Qiagen, Germantown, MD, United States) to 4 parts CFE for 2 h with DmrA-H_4_ or 1 h with H_6_-DfrA, H_6_-DfrB, Hni-DAGK-H_6_, and Mno-DAGK-H_6_. The CFE-NiNTA slurry was poured into a 10-ml polypropylene column (Bio-Rad Laboratories, Inc., Hercules, CA, United States) and washed three times with 5 ml of 50 mM Tris pH 8, 200 mM NaCl, 30 mM imidazole, 15 mM 2-mercaptoethanol (2-ME). Elution buffers consisted of 50 mM Tris pH 8, 200 mM NaCl, 15 mM 2-ME with 100 mM imidazole or 250 mM imidazole. Buffers were added to the column at room temperature (approximately 23°C) to minimize fluctuations in pH within the column.

Protein concentrations were determined by the Bradford procedure (Bradford, 1976) using bovine serum albumin (Pierce Biotechnology, Rockford, IL, United States) as the standard. The efficiency of protein purification and protein purity were analyzed using sodium dodecyl sulfate-polyacrylamide gel electrophoresis (SDS-PAGE) stained with Coomassie brilliant blue G-250 (Bio-Rad, Hercules, CA, United States) (Garfin, 1990). All histidine-tagged proteins were shown to be greater than 95% pure.

### Preparation of Dihydrosarcinapterin (H_2_SPT) From Methanogen Cell Extract

The H_4_MPT analog tetrahydrosarcinapterin (H_4_SPT) was obtained from the methanogen *Methanosarcina thermophila* TM-1 grown on acetate (Scott and Rasche, 2002) and purified by a previously developed method (Caccamo et al., 2004). Approximately 5 g of cells were removed from liquid nitrogen and sealed in a 37-ml amber anaerobic vial. Cells were purged with hydrogen gas for 10 min and transferred to an anaerobic chamber (Coy Products, Inc., Grass Lake, MI, United States) in 97% nitrogen and 3% hydrogen (Praxair, Inc., Danbury, CT, United States). An anoxic solution (10 ml) of 30 mM sodium acetate pH 4.3 and 200 mM 2-ME was added to re-suspend the cells. The vial containing the cells was sealed with a rubber stopper and aluminum crimp seal, boiled for 15 min (Precision Scientific, Chicago, MI, United States), allowed to cool, and then transferred into the anaerobic chamber. The boiled cell lysate was transferred in aliquots (1 mL) into 2-ml microcentrifuge tubes and centrifuged for 20 min at 13,000 ×*g* (Eppendorf Minispin plus, Hauppauge, NY, United States). During centrifugation, a 2-ml column of Sephadex A-25 diethylaminoethane (DEAE) was prepared in a 10-ml polypropylene column (Bio-Rad Laboratories, Inc., Hercules, CA, United States) and equilibrated with two column volumes of 50 mM MOPS, 1 M NaCl, 150 mM 2-ME [pH 6.8], followed by two column volumes of 50 mM MOPS, 150 mM 2-ME [pH 6.8]. The column was wrapped in aluminum foil to limit light exposure within the column. An aliquot of boiled CFE (200 μL) was removed for use in methylene-H_4_MPT reductase (MtdB) assays. The remaining boiled CFE was mixed with one volume of 50 mM MOPS, pH 6.8, 150 mM 2-ME. This mixture was added to the DEAE column. Fractions were collected from a step gradient of 50 mM MOPS, pH 6.8, 150 mM 2-ME with 0–1.0 M NaCl. Two 2-ml aliquots per NaCl step (0, 200, 400, 500, 600, and 1,000 mM) were collected. The highest concentration of H_4_SPT was found in the first 500 mM fraction of NaCl, as determined by the MtdB enzymatic assay (Rasche et al., 2004). Fractions were sealed in 10-ml anaerobic vials wrapped in foil and stored at -80°C.

To partially oxidize the H_4_SPT to H_2_SPT, the first 500 mM NaCl DEAE fraction or second 400 mM NaCl DEAE fraction was exposed to air for 100 s, by gently swirling for 80 s (approximately 2 swirls/s) at 30-s intervals. Oxidation of H_4_SPT was followed monitoring the increase in absorbance at 280 and 342 nm (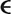
342_[Methanopterin]_ = 7.4 mM^-1^cm^-1^) (van Beelen et al., 1984) and the decrease in absorbance at 302 nm (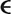
302_[H4MPT]_ = 15.2 mM^-1^cm^-1^) (Escalante-Semerena et al., 1984). Enzymatic assays were used to monitor levels of H_4_SPT (Rasche et al., 2004) and H_2_SPT (Caccamo et al., 2004) in addition to the wavelengths mentioned above. The oxidized 500 mM^-1^ fraction was transferred into an anaerobic chamber and aliquoted (100 μL) into 0.5-ml microcentrifuge tubes and sealed in a 10-ml anaerobic vial wrapped in aluminum foil. Aliquots were stored at -80°C.

### Dfr Assay

Reactions were prepared in an anaerobic chamber (97% N_2_ and 3% H_2_) in sealed 2-ml quartz masked cuvettes (Starna, Atascadero, CA, United States). The initial reaction mixtures (1 ml) consisted of about 3.6 μg of protein in an anoxic solution of 500 mM Tris (pH 7.5), 20 mM sodium ascorbate, 15 mM 2-ME, 50 μM H_2_F, and 0.1 mM NADPH. The reaction was initiated with the injection of protein using a 25-μL gas-tight syringe (Hamilton, Reno, NV, United States) that was purged with anoxic double-deionized water containing 20 mM 2-ME. The cuvette was gently inverted and placed back into the spectrophotometer. The oxidation of NADPH was monitored at 340 nm on a DU-800 spectrophotometer (Beckman Coulter, Brea, CA, United States) using a combined extinction coefficient for NADPH and H_4_F (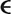
340_[NADPH__+__H4F_] of 12.3 mM^-1^cm^-1^. The effect of pH was analyzed as described above in 200 mM sodium phosphate for pH levels 5.8–8.0 and 200 mM sodium acetate buffer for pH 5.3. The effect of temperatures were tested over the range from 15 to 37°C. The cuvettes were covered and equilibrated in a water bath for 10 min at varying temperatures prior to addition of protein.

### DmrA Assay

The DmrA assay of Caccamo et al. (2004) was used based on modifications to a Dfr assay. Reactions were prepared in an anaerobic chamber in sealed 2-ml quartz masked cuvettes. The reaction mixture (250 μL or 1 ml) consisted of about 3.6 μg of enzyme in an anoxic solution containing 500 mM sodium acetate (pH 5.3), 20 mM sodium ascorbate, 1 mM EDTA, 15 mM 2-ME, 80 μM H_2_SPT, and 0.1 mM NADPH. The reaction was initiated with the injection of protein with a 25-μL gas-tight syringe, purged with anoxic double-deionized water containing 20 mM 2-ME. The cuvette was gently inverted and placed back into the spectrophotometer. The oxidation of NADPH was monitored at 340 nm (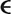
340_[NADPH]_ = 6.22 mM^-1^cm^-1^) (Dawson, 1986) on a DU-800 spectrophotometer.

### Specific Activity for Dfr, DmrA, and DAGK Assays and Kinetics Analysis for DmrA Assays

Rate calculations using the molar extinction coefficient for NAD(P)H were used to measure specific activity, where 1 unit is defined as 1 μmol of NAD(P)H oxidized per min per mg of protein for all assays. Enzyme kinetic constants (*K*_m_ and *V*_max_ values) were determined with a non-linear regression model fit to the Michaelis-Menten equation using GraphPad Prism v7.03 for Windows (GraphPad Software, La Jolla, CA, United States ^1^)

### DAGK Assay

Diacylglycerol kinases activity was assayed by coupling the oxidation of NADH to the production of phosphatidic acid (Badola and Sanders, 1997) (**Figure 1B**). The headspace of the DAG analog substrate (1,2-dihexanoyl-*sn*-glycerol in 50% ethanol) was purged under a gentle stream of nitrogen to evaporate the ethanol solvent until an oil residue remained. The sealed residue was transferred to an anaerobic chamber and reconstituted in an anoxic solution of 60 mM PIPES, 50 mM LiCl, 0.1 mM EDTA, 0.1 mM EGTA [pH 6.8] (150-μL) to a final concentration of 50 mg/ml (the approximate solubility of 1,2-dihexanoyl-*sn*-glycerol in phosphate buffered saline (PBS), pH 7.2.) Lactate dehydrogenase (LDH) (Roche, Mannheim, Germany), pyruvate kinase (PK) (Sigma-Aldrich, St. Louis, MO, United States), DAGK from *Escherichia coli* (Enzo Life Sciences, Farmingdale, NY, United States), and annotated DAGK from *M. nodulans* and *H. nitrativorans* were prepared by transferring 100 μL of each enzyme to 3-ml anaerobic vials and purging the headspace with a gentle stream of nitrogen for approximately 5 min on ice. The reaction was initiated with approximately 3.6 μg of protein in an anoxic reaction mixture (60 mM PIPES, pH 6.8, 50 mM LiCl, 0.1 mM EDTA, 0.1 mM EGTA), 1 mM phosphoenolpyruvate, 3 mM ATP, 2.6 mM 1,2-dihexanoyl-*sn*-glycerol, 20 mM magnesium acetate, 0.1 mM NADH, and 20 units each of LDH and PK. The oxidation of NADH was monitored using the molar absorption coefficient at 340 nm on a DU-800 spectrophotometer.

### Protein Computational Modeling

Conformational modeling of DmrA and DfrB was performed by Andrew Orry (Molsoft, San Diego, CA, United States) using the ICM package. The modeling template was the crystal structure of *Mycobacterium avium* dihydrofolate reductase co-crystallized with NADPH and trimethoprim (pdb 2w3v). The modeling method is based on the Internal Coordinates (IC) representation of molecular objects, which naturally reflects covalent bond geometry of molecule (Abagyan and Totrov, 1994; Abagyan et al., 1994). After initial placement of the aligned polypeptide chain onto the template structure, the side-chain torsion angles were predicted by simultaneous global optimization of the energy for all non-identical residues. Conformational modeling of protein side chains and loops involved internal coordinate definition of the molecular object combined with computationally efficient ICM Biased Probability Monte Carlo (BPMC) optimization. Optimization of the structures were done in an extended force field (Arnautova et al., 2011), which includes surface terms, electrostatics, and side chain entropy terms. The quality of the 3D model was assessed by an ICM procedure called Protein Health.

## Results

In *M. extorquens* AM1, three genes sharing similarity to dihydrofolate reductase (*dfrA, dfrB*, and *dmrA*) have been previously identified (Marx et al., 2003). Prior to the current work, only the protein encoded by the (*dmrA*) gene had been characterized biochemically (Caccamo et al., 2004; Rasche et al., 2004). In the current study, we used a bioinformatics approach to assess phylogenetic relationships among *M. extorquens* DfrA, DfrB, and DmrA, and homologs from other organisms. We also employed enzyme assays to assess the biochemical activities of DfrA, DfrB, DmrA, and two DmrA orthologs currently annotated as DAGKs.

### Sequence Alignment Searches of DfrB Orthologs Resulted in Three Distinct Clades

DfrB orthologs obtained in a BLASTx search from a non-redundant database in NCBI were used to construct an unrooted phylogenetic tree using a maximum-likelihood method with bootstrap analyses (Dawson, 1986; Kumar et al., 2016) (**Figure 2**). The resulting tree yielded three clades. Each clade contained either DfrA, DfrB, or DmrA from *M. extorquens* AM1. The sequences surrounding DfrA were from either Euryarchaeota, Plantomycete, Proteobacteria, or a Firmicute. The small clade containing DfrB revealed sequences from Proteobacteria and Actinobacteria. The largest clade (DmrA) contained homologs from Proteobacteria and a Firmicute.

### DfrA Is Closely Related to Annotated Dfr Orthologs From Bacteria

In the phylogenetic tree, the *M. extorquens* DfrA sequence was located among orthologs from planctomycetes and β-, γ-, and δ-proteobacteria (**Figure 2**). Two of the proteins in the DfrA clade have been previously crystallized as dihydrofolate reductases: a DfrA homolog from *Moritella profunda* (Mpr-Dfr) (Hay et al., 2009), and a trimethoprim-resistant ortholog from *E. coli* (Eco-DfrA24). This observation provides support for the hypothesis that *M. extorquens* DfrA may function as a standard dihydrofolate reductase All planctomycete homologs in the phylogenetic tree grouped with DfrA. Interestingly, one planctomycete sequence was annotated as a DAGK (TMED84-DK, **Figure 2**) in the DfrA clade. However, this ortholog was found to be only 14% identical to a known DAGK from *E. coli* using a percent identity matrix generated in Clustal Omega (Sievers et al., 2011; McWilliam et al., 2013; Li et al., 2015).

To further investigate connections to folate metabolism, genes in the neighborhood of *dfrA* were analyzed using the STRING v10.5 database, and a gene/protein interaction network module was constructed with *dfrA* biosynthesis (data not shown). In various genomes, genes with connection to *dfrA* included *thyA, folC, fhs, gcvT, glyA, metH, purH, purN, fmt*, and MexAM1_META1p0830 (*fmt-*like), many of which are associated with folate-requiring pathways of coenzyme, amino acid synthesis, and purine.

### One Clade Contained a Small Group of Orthologs Sharing 45–95% Identity With DfrB

Out of the vast number of DfrB orthologs identified by the BLAST search, only five sequences grouped tightly with and are closely related to *M. extorquens* DfrB in the phylogenetic tree (**Figure 2**). One ortholog was from an actinobacterium (*Streptomyces purpurogeneiscleroticus*) and the remaining were from α-proteobacteria. Of these five, two orthologs were annotated as Dfr and the remaining three were labeled as hypothetical proteins. The highest identity to DfrB (95%) was an ortholog annotated as Dfr from *M. populi* (Mpo-DfrB, **Figure 2**). This organism has been renamed as *M. extorquens* strain BJ001 (Marx et al., 2012).

Of the orthologs in the DfrB clade, only DfrB was located on a plasmid. Two genes located upstream of DfrB were a putative transposase and a protein of unknown function. The highest confidence for a homology match in the Phyre2 database for the protein of unknown function resulted in a riboflavin synthase domain-like superfamily (ferredoxin reductase FAD-binding domain-like family), with a reductase/isomerase/elongation factor common domain (30% identity, coverage).

### The DmrA Clade Included Annotated DAGKs From Various Bacteria

Homologs in the DmrA clade had identities ranging from 60 to 99% when compared to DmrA from *M. extorquens* AM1. The DmrA clade contained three homologs from α-proteobacteria annotated as dihydrofolate reductases (Dfr) (*M. mesophililcum, Granulibacter bethesdensis* CGDNIH1, and *M. populi*), and a large number of homologs annotated as DAGKs. Many of the annotated DAGKs contained amino acid regions predicted in NCBI to reduce dihydrofolate to H_4_F using NADPH as a cofactor. Thus, we tested whether some of these annotated DAGKs might function as dihydrofolate reductases or DmrA enzymes.

Most of the putative dihydromethanopterin reductases were from α-proteobacteria. The exceptions were two sequences from the β-proteobacteria *Azohydromonas australica* and *A. lata*) (respectively, 67 and 65% identity to *M. extorquens* DmrA). This is interesting because it is the first evidence of DmrA homologs in β-proteobacteria. In other β-proteobacteria, the proposed dihydromethanopterin reductases are not homologous to DmrA but instead resemble an archaeoflavoprotein (AfpA) found to restore a C_1_ growth phenotype in *M. extorquens* following *dmrA* knockout and complementation (Kalyuzhnaya et al., 2005). The AfpA group in β-proteobacteria has been renamed as dihydromethanopterin reductase B (DmrB). The crystal structure of DmrB points to the role of FMN cofactors in electron or hydride transfer to H_2_MPT (McNamara et al., 2014). It is intriguing that *A. australica* and *A. lata* contain homologs of both DmrA (**Figure 2**) and DmrB (with identities of 69 and 68%, respectively, to *Burkholderia xenovorans* DmrB). This raises the evolutionary question of why both forms of dihydromethanopterin reductase (DmrA and DmrB) might coexist in these organisms.

### *M. extorquens* DfrA and DfrB Enzyme Activities

**FIGURE 3:**
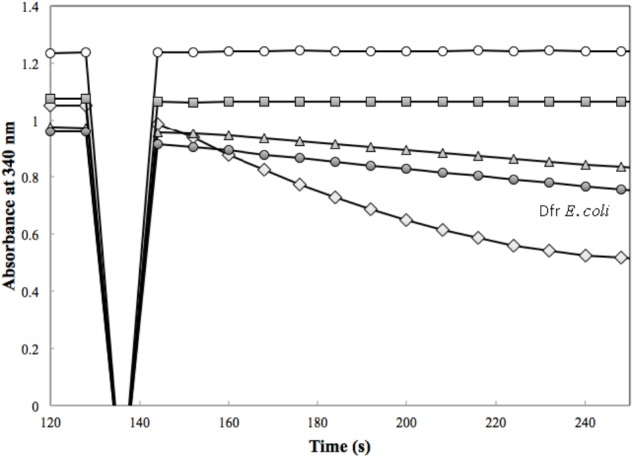
Dfr activity was assessed in the presence of 50 μM H_2_F with 1.7 μg of H_6_-DfrA (open diamond), 1.9 μg of H_6_-DfrB (triangle), 3.6 μg of Mno-DAGK-H_6_ (open circle), 3.6 μg of Hni-DAGK-H_6_ (square), and 1.0 μg of Dfr from *Escherichia coli* (closed circle).

**FIGURE 4:**
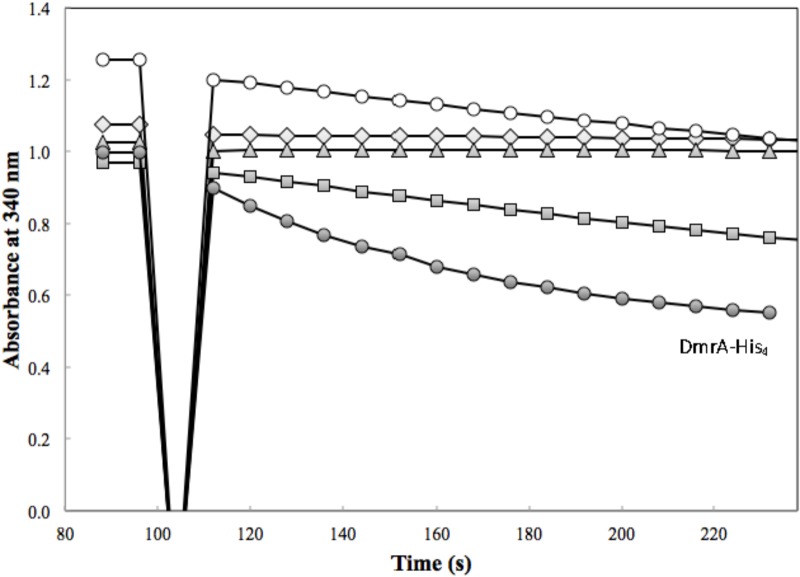
DmrA activity was assessed in the presence of 83 μM H_2_SPT with 3.4 μg of H_6_-DfrA (open diamond), 4.1 μg of H_6_-DfrB (triangle), 3.6 μg of Mno-DAGK-H_6_ (open circle), 0.72 μg of Hni-DAGK-H_6_ (square), and 4.3 μg of DmrA-H_4_ (closed circle).

To test the hypotheses that DfrA and DfrB function as dihydrofolate reductases, the enzymes were initially assayed in the presence of 50 μM H_2_F. Under these initial conditions, H_6_-DfrA and H_6_-DfrB reduced H_2_F with specific activities of 18.5 and 3.13 U/mg, respectively (**Figure 3** and **Table 1**). These values were within 2.5-fold of the rate obtained using a known dihydrofolate reductase from *E. coli* (7.3 U/mg) (**Figure 3** and **Table 1**). When DfrA and DfrB were tested for dihydromethanopterin reductase activity, only a trace of H_2_SPT reduction activity was observed for both enzymes (**Figure 4**, diamond and triangle; **Table 1**, column 3). This activity was only about 1% of the H_2_SPT reduction activity of DmrA-H_4_ measured at pH 5.3 (**Table 1**). Some caution should be taken in interpreting these data due to the histidine tags, which lacked a protease cut site and could not be removed. However, since the activities of H_6_-DfrA and H_6_-DfrB resembled that of untagged DfrB from *E. coli*, these data provide biochemical support that DfrA and DfrB are likely to function in converting dihydrofolate to H_4_F in *M. extorquens* cells.

**Table 1:** Initial tests of enzyme activity in the presence of pterin and DAG substrates.

Protein	Specific activity H_2_F (U/mg)	Specific activity H_2_SPT (U/mg)	Specific activity DAG (U/mg)
Eco-Dfr	7.7 ± 0.52^4^	—^a^	—^a^
H_6_-DfrA	18.5 ± 0.52^3^	0.041	—^a^
H_6_-DfrB	3.13 ± 0.60^3^	0.011	—^a^
DmrA-H_4_	—^a^	2.24 ± 0.26^3^	—^a^
Mno-DAGK-H_6_	None detected	0.63 ± 0.34^3^	0.43
Hni-DAGK-H_6_	None detected	2.82 ± 0.28^6^	0.55
Eco-DAGK	—^a^	—^a^	22.4


*^a^Not determined. Superscripts 3–6 denote number of replicates. For “no activity detected,” the limit of detection was 0.25 mg/ml.*

The effect of pH, temperature, and enzyme concentration were studied for H_6_-DfrA and H_6_-DfrB in preparation for kinetics studies. Over the pH range tested (5.3–8.0), the highest reaction rates for both enzymes were obtained from pH 6.8 to 7.0 (data not shown). For the temperatures tested (15–37°C or 40°C), H_6_-DfrA showed a broad temperature optimum from about 23–40°C, while H_6_-DfrB showed near constant reaction rates between 15 and 37°C. Thus, pH 6.8 and room temperature were used for kinetics measurements. DfrA activity showed a linear response to increasing enzyme concentration up to 1.8 μg per assay (0.093 μM H_6_-DfrA), while DfrB activity was linear up to 3.6 μg per assay (0.18 μM H_6_-DfrB).

**FIGURE 5:**
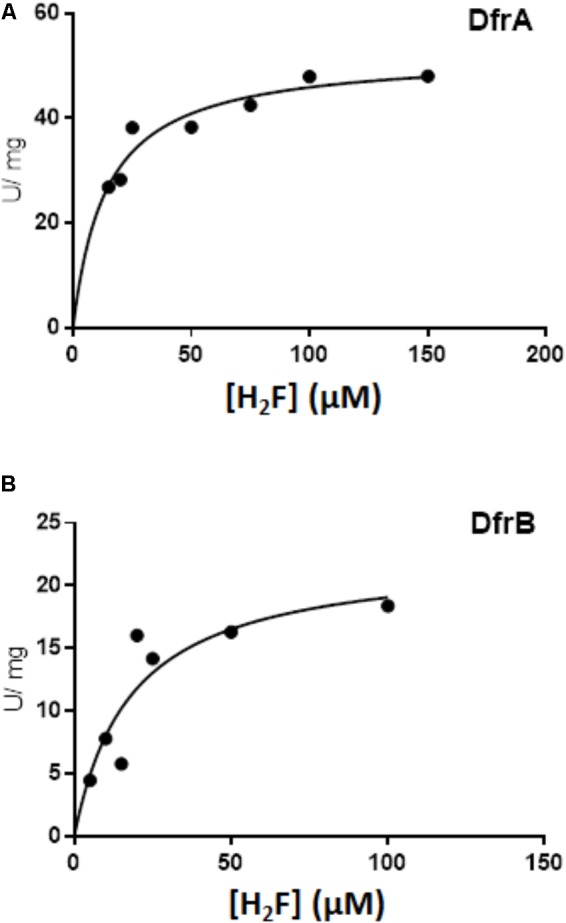
Kinetics of DfrA and DfrB with dihydrofolate as the substrate. Dihydrofolate reductase activity was measured as described in Section “Materials and Methods” in the presence of 100 μM NADPH and either DfrA **(A)** or DfrB **(B)**. Data were fit to the equation for a hyperbola using non-linear least-squares fit with GraphPad Prism 7.04 Software. The estimated *K*_M_ and *V*_max_ values are presented in the Section “Results.”

To estimate kinetic values, the concentration of dihydrofolate was tested over the range from 0 to 150 μM (**Figure 5**). When fit to the Michaelis-Menten equation for a hyperbola, the estimated *K*_M_ values were similar (14 ± 3.0 μM dihydrofolate for DfrA and 18 ± 8.9 μM for DfrB). The estimated *V*_max_ for DfrA was 52 ± 2.8 U/mg, corresponding to a *k*_cat_ of 17/s. The *V*_max_ for DfrB was about 2.5 times lower (22.5 ± 4.2 U/mg, *k*_cat_ of 7.2/s).

### Enzyme Activity Assays for Orthologs Within the DmrA Clade

The activity of *M. extorquens* DmrA-H_4_ was compared with two annotated DAGKs sharing different degrees of identity with *M. extorquens* DmrA. The *M. nodulans* homolog (Mno-DAGK- H_6_) was 84% identical to DmrA, and the *H. nitrativorans* homolog (Hni-DAGK-H_6_) was 69% identical to DmrA. We first tested whether these enzymes showed NADPH-dependent dihydrofolate reductase activity, but no activity was detected with the addition of either the Mno or Hni enzyme (**Figure 3** and **Table 1**).

Annotated DAGKs from *M. nodulans* and *H. nitrativorans* were both capable of reducing H_2_SPT (**Figure 4**). Under the initial screening conditions, the specific activity of the Hni-DAGK-H_6_ was about the same as that of DmrA-H_4_, while the rate for Mno-DAGK-H_6_ was 3–4 times lower (**Table 1**). The lower activity of Mno-DAGK-H_6_ in the initial screening studies may be explained by the higher *K*_M_ values obtained later in the kinetics studies (**Table 2**). The Mno-DAGK-H_6_ appeared to have lower affinity for H_2_SPT (apparent *K*_M_ of 695 μM H_2_SPT) compared to the apparent *K*_M_ values for *M. extorquens* DmrA-H_4_ and Hni-DAGK-H_6_ (193 and 102 μM H_2_SPT, respectively). Despite the differences in *K*_M_ values, the *V*_max_ estimates for the three enzymes were similar, differing only by a factor of two (**Table 2**).

**Table 2:** Kinetic values (apparent *K*_M_, *V*_max_, and *k*_cat_) for DmrA and DmrA orthologs.

Protein	*K*_M(app)_	*V*_max_ (U/mg)	*k*_cat_(s^-1^)
*M. extorquens* AM1 DmrA-H_4_	193 ± 71^4^	5.72 ± 1.2^4^	1.5
Mno-DAGK-H_6_	695 ± 176^3^	5.94 ± 2.6^3^	1.6
Hni-DAGK-H_6_	102 ± 73^2^	10.9 ± 1.8^2^	2.9


*Superscripts 2–4 denote number of replicates.*

### DAGK Assays for Homologs Within the DmrA Clade

**FIGURE 6:**
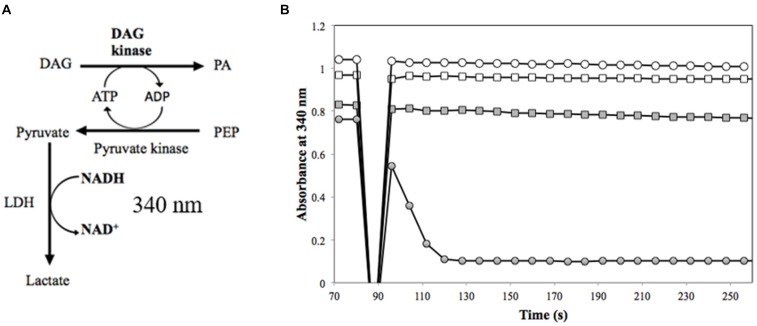
Measurement of DAGK activity. **(A)** In the DAGK assay (Badola and Sanders, 1997), the conversion of DAG to phosphatidic acid (PA) produces ADP, which is used in the pyruvate kinase reaction to convert phosphoenolpyruvate (PEP) to pyruvate. This reaction is coupled to the reduction of pyruvate to lactate by lactate dehydrogenase (LDH). The oxidation of NADH is monitored as a loss in absorbance at 340 nm for DAGK activity. There is a 1:1 ratio of one mole of PA produced to one mole NADH oxidized. **(B)** DAGK activity of 3.5 μg of Mno-DAGK-H_6_ (open circle), 3.6 μg of Hni-DAGK-H_6_ (square), boiled Hni-DAGK-H_6_ (closed square) compared to DAGK control from *E. coli* (closed circle).

To test the alternative hypothesis that the DmrA homologs might contain the annotated DAGK activity, a modified DAGK assay was performed (**Figure 6A**). In these studies, the DAG analog, 1,2-dihexanoyl-*sn*-glycerol was used, but β-octyl glucoside (OG) and dimyristoyl phosphatidylcholine (DMPC) were excluded. To show that the modified assay was functioning properly in our lab, DAGK from *E. coli* was tested as a control (**Figure 6B**). Under the conditions used, the specific activity of the *E. coli* enzyme (1 μg of protein) was 24.2 U/mg (**Table 1**), which is comparable to the published value of 22.0 U/mg (Badola and Sanders, 1997).

In the DAGK assay, the addition of 3.5 μg of Mno-DAGK-H_6_ or Hni-DAGK-H_6_ produced specific activities of 0.43 and 0.55 U/mg, respectively (**Figure 6B** and **Table 1**). This rate was only 1–2% of the activity of commercially purchased Eco-DAGK when comparable amounts of enzyme were used. The slow rate of Hni-DAGK-H_6_ activity proceeded constantly over a course of 5 min, in contrast with that of the boiled enzyme control, which yielded no activity (**Figure 6B**).

## Discussion

The results of the current study may be interpreted in the context of the previously published model predicting that the *M. extorquens* DmrA protein evolved from an ancestral dihydrofolate reductase (Dfr) following transfer of H_4_MPT biosynthesis genes from archaea to bacteria (Marx et al., 2003). This hypothesis is based on the sequence similarity of DmrA to known dihydrofolate reductase sequences, combined with the observation that disruption of the *M. extorquens dmrA* gene produces a phenotype similar to that of deletion mutants in H_4_MPT biosynthesis genes (Marx et al., 2003; Rasche et al., 2004; Chistoserdova et al., 2005). Due to the absence of archaeal redox cofactors in bacteria, archaeal oxidoreductases like dihydromethanopterin reductases may have been non-functional in bacteria. To resolve this issue, two separate lineages of bacterial dihydromethanopterin reductases appear to have evolved: one of bacterial origin (DmrA) found almost exclusively in α-proteobacteria, and a second lineage (AfpA/DmrB) derived from an archaeal flavoprotein called DmrX.

The results of the current study are consistent with a bacterial origin for DmrA in α-proteobacteria. The phylogenetic tree in **Figure 2** places *M. extorquens* DfrA, DfrB, and DmrA in separate clades. Duplication of a *dfr* gene followed by mutations that changed specificity for the pterin substrate would account for the presence of both dihydrofolate reductase and dihydromethanopterin reductase activities in extant α-proteobacteria (**Table 1**).

*Methylobacterium extorquens* DfrA has been proposed to function as a standard dihydrofolate reductase based on co-localization of *dfrA* with genes encoding H_4_F biosynthesis and H_4_F-dependent enzymes and additional gene neighborhood analysis of multiple genomes (Chistoserdova et al., 2003; Marx et al., 2003; this study). Prokaryotic genes of related functions often occur together in operons or gene clusters, as demonstrated by the large cluster of proteobacterial genes related to H_4_MPT-dependent metabolism (Chistoserdova et al., 1998; Kalyuzhnaya et al., 2005). In the current study, the dihydrofolate reductase activities of DfrA and DfrB were demonstrated biochemically for the first time and were comparable to the activity of a known Dfr from *E. coli* (**Table 1** and **Figure 5**). The evolutionary potential for altering substrate specificity from dihydrofolate to dihydromethanopterin is also supported to some extent by enzymatic assays in which traces of H_2_SPT reduction activity were detected (**Table 1**). Conversely, *M. extorquens* DmrA has been shown to reduce H_2_SPT at relatively high rates and dihydrofolate at low rates (Caccamo et al., 2004), possibly representing a vestige of an ancestral dihydrofolate reductase activity.

**FIGURE 7:**
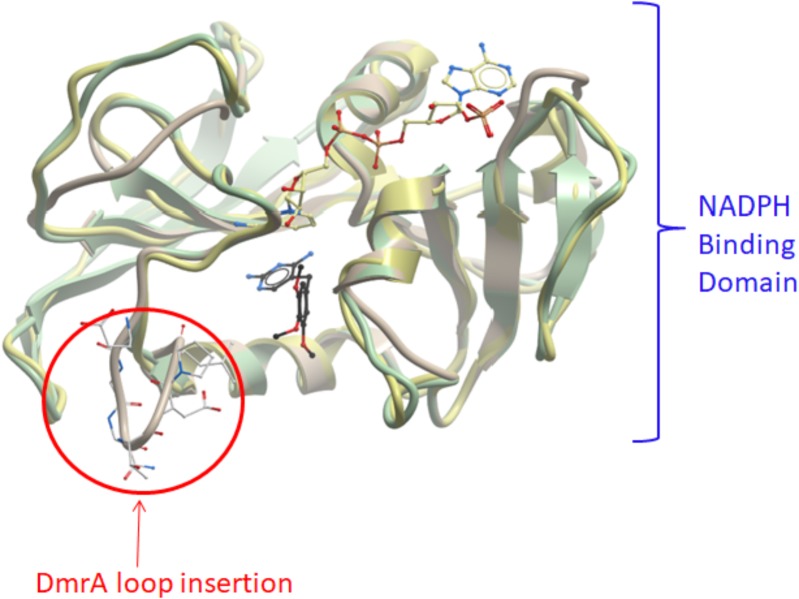
Computational models of DmrA and DfrB. 3D models of DmrA and DfrB were constructed at described in Section “Materials and Methods” using ICM-Pro (Molsoft, San Diego, CA, United States) with *Mycobacterium avium* dihydrofolate reductase as the template (pdb 2w3v). The NADPH binding sites appeared conserved between the two protein models (blue label) with the position of NADPH predicted by analogy to the template structure. An insertion of 7 amino acids in DmrA (residues 25–31) formed a unique loop (circled in red). The DmrA model also predicted the deletion of two C-terminal β-strands found in both DfrB and the template.

Protein computational modeling also demonstrates the potential for changing the specificity of dihydrofolate reductase toward affinity for dihydromethanopterin. Molecular models of *M. extorquens* DmrA and DfrB were constructed by Andrew Orry (Molsoft L.L.C., San Diego, CA, United States) (**Figure 7**) and predict that DmrA (**Figure 7**, yellow ribbon structure) and DfrB (green ribbon structure) share a similar overall protein fold consisting of primarily parallel β-sheets connected by α-helices. In particular, secondary structural features are conserved in the NADPH binding domain, which includes DmrA residues 59 to 85. This would account for the conserved use of NADPH as an electron donor by both DmrA and DfrA. Unique structural features of DmrA occur in the active site region distant from the NADPH binding domain, where the pterin substrate is presumed to bind. The DmrA model shows an insertion of 7 amino acids (residues 25–31) forming a loop that is absent in the models of DfrB and *Mycobacterium* dihydrofolate reductase (**Figure 7**). Another difference is that DmrA also lacks two of the C-terminal β-strands found in the dihydrofolate reductase structures. Although the DmrA model could not predict the details of the DmrA pterin binding site with confidence, the insertion of a DmrA loop and the loss of two β-strands over evolutionary time might have served to accommodate the structural differences between dihydromethanopterin and dihydrofolate. A crystal structure of DmrA would be needed to create a detailed model of the dihydromethanopterin binding site.

The function of the second dihydrofolate reductase in *M. extorquens* (DfrB) remains a mystery. The estimated *K*_M_ values for DfrA and DfrB were similar, while the *V*_max_ for DfrB appeared lower than that of DfrA (**Figure 5**). The presence of a second Dfr is not uncommon in bacteria. Multiple copies of dihydrofolate reductase can provide varying sensitivities to folate competitors such as trimethoprim (Huovinen, 1987). The *M. extorquens* *dfrB* gene is located on a megaplasmid, and the protein has 47% similarity to a trimethoprim-resistant dihydrofolate reductase encoded on *E. coli* plasmid *pCJ001*, leading to a hypothesis for a role of DfrB in antimicrobial resistance (Jansson and Skold, 1991; Marx et al., 2003). Another proposed role for *M. extorquens* DfrB as an intermediate in the evolution of DmrA may be inferred from the closer sequence identity between DfrB and DmrA (34% identical) compared that of DfrA (26% identical to DmrA) (Marx et al., 2003) and the phylogenetic position of the DfrB clade between DfrA and DmrA (**Figure 2**). While this might be possible, the similar low rates of H_2_SPT reduction by DfrA and DfrB (**Table 1**) do not seem to favor DfrB as a preferred intermediate in the evolution toward DmrA.

For the DmrA clade, the current annotation of many orthologs as DAGKs was surprising based on sequence alignments. For example, while the annotated DAGKs from *M. nodulans* and *H. nitrativorans* are 84 and 69% identical to *M. extorquens* DmrA, they share only 15 and 12% identity, respectively, with a known DAGK from *E. coli*. Dihydromethanopterin reductase activity measured at pH 5.3 was observed for Mno-DAGK-H_6_ and Hni-DAG-kinase-H_6_ (**Figure 4** and **Tables 1**, **2**), while dihydrofolate reductase activity was not detectable for either enzyme under the conditions studied (**Table 1** and **Figure 3**). The DAGK activities of the two enzymes were also very low compared to the activity of a known *E. coli* DAGK under the same conditions (1–2%) (**Table 1** and **Figure 6**). At this time, we cannot rule out the possibility that the annotated DAGKs in this study may be bifunctional enzymes with both DmrA and DAGK activities playing a role in methylotroph cells. However, given the low sequence identity to characterized DAGKs, the low DAGK activities (**Figure 6**), and kinetics values similar to those of DmrA (**Table 2**), we propose that DAGKs sharing at least 69% identity with *M. extorquens* AM1 DmrA should be renamed as dihydromethanopterin reductases.

An explanation for the large apparent *K*_M_ difference between Mno-DAGK-H_6_ and DmrA-H_4_ might be attributed to either the protein structural health following purification through nickel affinity chromatography or the physical and chemical environment in which *M. nodulans* is found in nature. *M. nodulans* exhibits both nitrogen-fixation and specific nodulation of *Crotalaria* species. These features have not been observed in the *Methylobacterium* species that have been tested thus far (Sy et al., 2001). In the nodule environment, high amounts of methanol and methylotrophic activity have been observed (Jourand et al., 2005). A high apparent *K*_M_ for H_2_MPT may enable *M. nodulans* to regulate dihydromethanopterin reductase activity to accommodate large influxes of methanol.

Another point of interest is the finding of a DmrA homolog in *A. lata* and *A. australica*. These two species of β-proteobacteria also contain an archaea-like dihydromethanopterin reductase with a redox-active FMN cofactor (AfpA/DmrB) (Ding and Ferry, 2004; Kalyuzhnaya et al., 2005). The presence of two phylogenetically diverse forms of dihydromethanopterin reductase (DmrA and DmrB) in a single organism invites additional studies of the evolutionary history and differential roles of the two enzymes in the C_1_ metabolism of these cells.

Methylotrophic microorganisms are valuable in biotechnology processes that use methanol as an alternative to sugars as a carbon substrate for the biosynthesis of industrial products such as biofuels and biopolymers (Schrader et al., 2009). Additional benefits include the potential to synthesize polyhydroxybutyrates and uncommon dicarboxylic acids or polyketides using the ethylmalonyl-CoA pathway of *Methylobacterium* species. The ability to grow on minimal media might also simplify product recovery compared to the separations required with rich media, such as Luria broth (Ochsner et al., 2015). Using a natural methylotroph, as opposed to bioengineering *E. coli*, could eliminate the need to engineer methods to alleviate a potential buildup of formaldehyde as a toxic intermediate during methanol oxidation. The relatively high *K*_M_ for DmrA (DAGK) from *M. nodulans* might allow responsiveness at higher concentrations of H_2_SPT to help accommodate increases in methanol concentration. The ability of methylotrophs to process large extracellular concentrations of methanol, combined with the metabolic machinery to transform chemicals while avoiding formaldehyde bioaccumulation, could provide advantages for methanol-based biotechnology in the future.

## Dataset Statement

All relevant data is either contained within the manuscript or will be made available by the authors, without undue reservation, to any qualified researcher.

## Author Contributions

MB designed the principle experiments based on an original research idea by CA. MB, CA, KW, and YM executed the experiments and interpreted the data. MB, YM, and MR prepared the manuscript.

## Conflict of Interest Statement

The authors declare that the research was conducted in the absence of any commercial or financial relationships that could be construed as a potential conflict of interest.

## Footnotes

1 www.Graphpad.com
